# Measurement Properties of the Pediatric Anesthesia Emergence Delirium Scale: A Confirmatory Factor Analysis‐Based Study

**DOI:** 10.1111/pan.15046

**Published:** 2024-11-29

**Authors:** Jenny Ringblom, Ingrid Wåhlin, Marie Proczkowska, Laura Korhonen, Kristofer Årestedt

**Affiliations:** ^1^ Center for Social and Affective Neuroscience and Department of Biomedical and Clinical Sciences Linköping University Linköping Sweden; ^2^ Department of Anesthesiology and Intensive Care Region Kalmar County Kalmar Sweden; ^3^ Faculty of Health and Life Sciences Linnaeus University Kalmar Sweden; ^4^ Department of Research Region Kalmar County Kalmar Sweden; ^5^ Child and Adolescent Department Psychiatric Clinic, Helgelandssykehuset Mo i Rana Norway; ^6^ Department of Child and Adolescent Psychiatry and Department of Biomedical and Clinical Sciences Linköping University Linköping Sweden; ^7^ Barnafrid Linköping University Linköping Sweden

**Keywords:** emergence delirium, instrument development, pediatric anesthesia, postoperative behavioral changes

## Abstract

**Background:**

Emergence delirium is a well‐known and common problem in children recovering from anesthesia. The five‐item Pediatric Anesthesia Emergence Delirium scale is one of the most commonly used instruments for assessing this condition, but the scale has been questioned regarding its latent structure, i.e., whether its items cover just one underlying construct. It has been suggested that the scale's first three items might identify emergence delirium better than the last two.

**Aim:**

The aim of this study was to evaluate the measurement properties of the Pediatric Anesthesia Emergence Delirium scale with a focus on its latent structure and cutoff scores, using appropriate statistical methods for ordinal data.

**Methods:**

A total of 350 children under 7 years of age, undergoing adenoidectomy, with or without additional tonsillotomy or minor procedures like paracentesis, tongue‐tie release, or cerumen removal, were enrolled in the study. At the recovery unit, emergence delirium and pain were registered.

**Results:**

The confirmatory factor analyses demonstrated that the two‐factor model, including *emergence delirium‐specific behaviors* (first three items) and *emergence delirium‐nonspecific behaviors* (last two items), established an excellent model fit according to the *χ* ^2^ goodness‐of‐fit statistics, Root Mean Square Error of Approximation, Comparative Fit Index, Tucker‐Lewis Index, and Standardized Root Mean Square Residual. The ordinal alpha of 0.98 and the ordinal omega of 0.96 supported the internal consistency reliability of the *emergence delirium‐specific behaviors.* The convergent validity was supported due to a strong correlation between *emergence delirium‐nonspecific behaviors* and the Face, Legs, Activity, Cry, and Consolability scale. The receiver‐operating characteristic curve analyses resulted in two tentative cutoff scores for *emergence delirium‐specific behaviors*¸ ≥ 6 and ≥ 8.

**Conclusion:**

The Pediatric Anesthesia Emergence Delirium scale's first three items are a more valid and reliable measure of emergence delirium than its original five items.

## 1 Introduction

Emergence delirium (ED) is a well‐known and common problem in children recovering from anesthesia. This complex condition has been described as characterizing non‐purposefulness; eyes averted, staring or closed; a thrashing behavior; and non‐responsivity [1, 2]. The behavior can last up to 45 min, but in a few cases has been described as lasting up to 2 days [2, 3]. It is of great importance to identify and prevent ED, as the child may cause him−/herself injury due to unintentional and irrational actions, extra nursing care is required and, additionally, it may cause hospital discharge to be delayed [4]. Furthermore, parents can experience the ED situation as incomprehensible, with feelings of fear, insecurity, powerlessness, and guilt [5]. In addition to these immediate behavioral changes, long‐term problems such as nightmares and separation anxiety have been reported, although research findings are inconclusive in this regard [6, 7].

Even if ED is described as a common problem in children, the incidence ranges broadly. A recent study, investigating children premedicated with dexmedetomidine for elective strabismus surgery, reported an incidence of 11% [8]. In contrast, a considerably higher frequency (80%) was observed in children undergoing ear, nose, and throat surgery who were anesthetized with desflurane [9]. There are several possible reasons for this variation, such as the age of the participating children, the type of surgical procedure performed, or the anesthesia method chosen [2, 10, 11]. Other explanations for the variation include the lack of a universally accepted definition of ED and the employment of a variety of non‐validated assessment tools [12], not aligned with the established fifth edition of Diagnostic and Statistical Manual of Mental Disorders (DSM‐V) criteria for diagnosing delirium [13]. These scales may be more indicative to agitation or pain, leading to confusion and inflated scores of ED [12].

The Pediatric Anesthesia Emergence Delirium (PAED) scale is a widely employed instrument and the only validated tool for quantifying the severity of ED in anesthetized children. The scale includes five items and has overall demonstrated satisfactory psychometric properties regarding validity and reliability [14, 15]. However, the scale has been questioned concerning its latent structure; i.e., whether its items cover just one underlying construct. Already when the instrument was being developed, its constructors pointed out that the first three items (ED I) might identify ED better than the last two (ED II). It has been suggested that these last two items reflect pain as well as ED [14, 16]. Two Italian studies have examined the sensitivity and specificity of this two‐dimensional PAED scale, and in both, ED I demonstrated higher sensitivity than ED II in identifying ED [16, 17]. In contrast to these findings, a psychometric evaluation study by Ringblom et al. supported the hypothesized latent structure in which all items are expected to reflect ED, i.e., unidimensionality. However, one important limitation of that study is that it was based on an exploratory factor analysis that did not take into consideration the ordinal nature of data [15]. Therefore, the latent structure needs to be further evaluated through a confirmatory approach, which is appropriate for ordinal data.

Furthermore, the PAED scale lacks an established optimal cutoff score for identifying ED. The scale's constructors recommended a threshold of ≥ 10 for medical treatment in ED [14]. Bajwa et al. [18] demonstrated that a score ≥ 13 provided the best sensitivity and specificity when compared with the assessment of an experienced pediatric anesthetist, but other cutoff scores have also been suggested, for example ≥ 9 [19] and ≥ 16 [20]. The recommended cutoff scores for ED I and ED II also differ across studies. In a study by Locatelli et al., the recommended cutoff scores are ≥ 9 for ED I and ≥ 5 for ED II [16]; in contrast, a study by Somani et al. suggests cutoff scores of ≥ 6 and ≥ 4 for ED I and ED II, respectively [17]. Thus, the cutoff scores for the PAED scale need to be further explored.

## 2 Aim

The aim of this study was to evaluate the measurement properties of the PAED scale with a focus on its latent structure and cutoff scores, using appropriate statistical methods for ordinal data.

## 3 Methods

### 3.1 Study Design

This methodological study was a part of a larger longitudinal multicenter study. In the present study, cross‐sectional data from the baseline assessment was used. Ethical approval was granted by the Regional Ethical Review Board in Linköping (No. 2015/195‐31, 2018/328‐32) and the Swedish Ethical Review Authority (No. 2019‐00351). Written informed consent was obtained from the parents or guardians of the participating children.

### 3.2 Participants and Procedure

The study was conducted at four Swedish county hospitals between November 2018 and January 2022. All children under 7 years of age who were scheduled for adenoidectomy, with or without additional tonsillotomy or minor procedures like paracentesis, tongue‐tie release, or cerumen removal, were consecutively invited to participate. Children of parents or guardians who lacked proficiency in Swedish were excluded. A designated nurse at each hospital identified children who fulfilled the inclusion criteria. The research team obtained the contact details for the parents or guardians and mailed them information about the study along with an invitation to participate. They consented to participation by returning a signed consent form via a prepaid envelope before surgery or in person on the day of surgery.

### 3.3 Data Collection

Data and behavioral observations were collected at three time points: before, during, and after surgery. Preoperative data included assessment of the child's personality traits and prior hospital experiences. The anesthetic managements were not standardized. The data employed in the present study encompassed behavioral observations conducted at the recovery unit. This data was collected by a registered nurse at the recovery unit, who assessed the child's behavior using the PAED scale and the Face, Legs, Activity, Cry, and Consolability (FLACC) scale, upon awakening or if the child was already awake upon arrival. The procedures employed for treating ED and pain were in accordance with local guidelines. Data concerning age, sex, and type of anesthesia was also collected.

#### 3.3.1 Pediatric Anesthesia Emergence Delirium (PAED) Scale

The PAED scale consists of the following items: (1) the child makes eye contact with the caregiver; (2) the child's actions are purposeful; (3) the child is aware of his/her surroundings; (4) the child is restless; and (5) the child is inconsolable. Each item is rated on a five‐point scale, ranging from “Not at all” (0) to “Extremely” (4), with the first three items reverse‐scored. The total score ranges from 0 to 20, with higher scores indicating greater severity of ED [14]. Permission to use the PAED scale in this study was obtained from its constructors.

#### 3.3.2 Face, Legs, Activity, Cry, and Consolability (FLACC) Scale

The FLACC scale is an observational instrument for identifying pain in children, in which the professionals rate behaviors within five categories: face, legs, activity, cry, and consolability. Each category is rated on a three‐point scale: no problems (0), moderate problems (1), and severe problems (2). The ratings are summarized in a total score with a possible range of 0 to 10, with high scores reflecting higher pain levels. The instrument has demonstrated satisfactory psychometric properties in international and Swedish studies including preschoolers/children of various ages [21, 22]. A license agreement for using the instrument was obtained from the University of Michigan, reference number 10523.

### 3.4 Statistical Analysis

Descriptive statistics were used to present the study variables: continuous data was described with means and standard deviations, ordered categories with medians and quartiles, and non‐ordered categorical data with frequencies.

Data quality was evaluated in terms of item and scale score distributions; descriptive statistics were used. Skewness statistics were also used: A normal distribution has a value close to 0. Inter‐item and item total correlations adjusted for overlaps were calculated using polychoric and polyserial correlations (*r*_pol_) to handle the ordinal nature of the data.

Confirmatory factor analysis (CFA) was used to evaluate the hypothesized factor structure, the original one‐factor model [14], and the suggested two‐factor model [16, 17]. The two‐factor model encompassed ED I and ED II. To handle the ordinal indicator variables (i.e., items with ordered categories), the weighted least squares mean, and variance adjusted (WLSMV) estimation method and a polychoric correlating matrix were used. Model fit was evaluated in terms of parameter estimates (e.g., strengths of factor loadings and absence of correlated error variances and cross‐loadings), absence of Haywood cases, and different goodness‐of‐fit indices to measure absolute fit (Standardized Root Mean Square Residual [SRMR]), parsimony correction fit (Root Mean Square Error of Approximation [RMSEA]), and comparative fit (Comparative Fit Index [CFI], and Tucker–Lewis Index [TLI]). A good model fit has RMSEA values close to or below 0.06, SRMR close to or below 0.08, and CFI and TLI close to or above 0.95. In addition, the residual covariance matrix and the modification index were examined for each CFA model [23].

Internal consistency reliability was evaluated using ordinal alpha, an ordinal version of Cronbach's alpha (α) and ordinal omega (ω).

To evaluate convergent validity, an aspect of construct validity, the PAED scale, ED I, and ED II were correlated with the FLACC scale using Spearman's correlation. A stronger correlation was hypothesized between ED II and the FLACC scale compared to that between the PAED scale as well as ED I and the FLACC scale.

Receiver‐operating characteristic (ROC) curve analyses were used to suggest a preliminary cutoff score of ED I. First, two classifier variables—i.e., binary grouping variables including children with and without ED—were created based on the two most common suggested cutoff scores of the original PAED scale, ≥ 10 [14] and ≥ 13 [18]. The analyses included the area under the curve (AUC), sensitivity, and 1‐specificity for the different possible cutoff scores of ED I, and the Youden index to estimate the optimal cutoff values.

The level of statistical significance was set at *p* < 0.05. All data analyses were conducted in R 4.3.2 (R Core Team, R Foundation for Statistical Computing, Vienna, Austria) including the following packages: correlation 0.8.4, cutpointr 1.1.2, lavaan 0.6–16, psych 2.3.9, semTools 0.5–6, and summarytools 1.0.1. The graphs for the ROC curves were created in Stata 18.0 (StataCorp LLC, College Station, TX, USA).

## 4 Results

### 4.1 Sample Characteristics

During the study period, 576 children were eligible for inclusion. Of these, the parents or guardians of 171 children declined participation and another 43 were not followed up by the healthcare professionals as intended. Additionally, 12 children had completely missing data on the PAED scale and were therefore excluded from the study. Thus, the final sample contained 350 children with a mean age of 3.8 (SD 1.5) years. Most of the children (46.0%) were anesthetized solely with propofol (Table 1).

**TABLE 1:** Sample characteristics (*n* = 350).

Age, mean (SD) [min–max]	3.8 (1.5) [1–6]
Sex, *n* (%)	
Boys	195 (55.7)
Girls	155 (44.3)
Premedication, *n* (%)	
Yes	75 (22.9)
No	253 (77.1)
Missing data	22
Anesthesia, *n* (%)	
Sevoflurane, solely	21 (6.7)
Propofol, solely	145 (46.0)
Induction Sevoflurane, maintenance Propofol	67 (21.3)
Induction Propofol, maintenance Sevoflurane	61 (19.4)
Induction Sevoflurane, maintenance Sevoflurane, ending Propofol	6 (1.9)
Induction Propofol, maintenance Sevoflurane, ending Propofol	15 (4.8)
Missing data	35
Pre‐ and intraoperative analgesics, *n* (%)	
Paracetamol	344 (98.3)
NSAIDs	280 (80.0)
Opioids	350 (100.0)
α2 agonists	219 (62.6)
PAED, Mdn (Q1–Q3) [min–max]	
Total score	3 (0–8) [0–20]
ED I	2 (0–6) [0–12]
ED II	0 (0–2) [0–8]
FLACC, Mdn (Q1–Q3) [min–max]	0 (0–3) [0–10]

Abbreviations: FLACC, Face, Legs, Activity, Cry, and Consolability scale; NSAIDs, non‐steroidal anti‐inflammatory drugs; PAED, Pediatric Anesthesia Emergence Delirium scale.

### 4.2 Item and Scale Score Statistics

Missing values were low and ranged from 0.3% to 0.6% (Table 2). These seven missing values were related to three observations. Thus, the share of computable scores without imputations was 99.1%.

**TABLE 2:** Item statistics and item‐total correlations (*n* = 350).

Items	Mdn (Q1, Q3)	Item score distribution, %^a^						ITC^c^	*α* ^d^
		0	1	2	3	4	Missing		
1. Eye contact^b^	0 (0, 2)	51.4	16.6	14.0	9.4	8.3	0.3	0.91	0.95
2. Purposeful actions^b^	1 (0, 2)	44.6	21.1	14.6	10.9	8.3	0.6	0.93	0.94
3. Aware of surroundings^b^	1 (0, 2)	43.7	22.9	14.3	12.6	6.3	0.3	0.92	0.95
4. Restless	0 (0, 1)	63.1	14.0	12.9	5.4	4.0	0.6	0.81	0.96
5. Inconsolable	0 (0, 1)	71.7	12.9	8.6	2.9	3.7	0.3	0.88	0.95

^a^ 0 = Not at all, 1 = Just a little, 2 = Quite a bit, 3 = Very much, 4 = Extremely.

^b^ Reversed scored items.

^c^ Polyserial item‐total correlations adjusted for overlaps.

^d^ Ordinal alpha if item deleted.

All items on the PAED scale had a positive skewed distribution with floor effects (Table 2). This was also shown for the scale scores; the skewness statistics were 1.11 for PAED, 0.85 for ED I, and 1.70 for ED II. The share of floor effects for the scales was 36.6% for PAED, 40.5% for ED I, and 60.9% for ED II.

### 4.3 Inter‐Item and Item Total Correlations

The inter‐item polychoric correlations varied between 0.72 and 0.96, with a mean correlation of 0.83. The strongest correlations were found between Items 1, 2, and 3 (*r*_pol_ 0.91–0.96) and between Items 4 and 5 (*r*_pol_ 0.88). Lower correlations were found between these two sets of items (*r*_pol_ 0.72–0.81) (Table 3). The item‐total polyserial correlations (ITC) varied between 0.81 and 0.93, with Items 4 and 5 having the lowest values. Ordinal alpha if item deleted ranged from 0.94 to 0.96 (Table 2).

**TABLE 3:** Inter‐item polychoric correlations, listwise deletion (*n* = 347).

	1	2	3	4	5
1. Eye contact	1.00				
2. Purposeful actions	0.94	1.00			
3. Aware of surroundings	0.91	0.96	1.00		
4. Restless	0.73	0.74	0.72	1.00	
5. Inconsolable	0.79	0.81	0.81	0.88	1.00

### 4.4 Factor Structure

The one‐factor model showed a poor model fit according to the *χ* ^2^ goodness‐of‐fit statistics and RMSEA, but a good fit according to CFI, TLI, and SRMR (Table 4). The factor loadings for the one‐factor model ranged between 0.86 and 0.99 (Table 5). No Haywood cases were detected, but the modification index and the residual covariance matrix indicated that the error variances between Items 4 and 5 were correlated. As these items have been questioned in previous research [16, 17] for not being delirium‐specific, and because they also showed the weakest inter‐item polyserial correlations (Table 3), a second modified two‐factor model was considered. This model, including the factors *emergence delirium‐specific behaviors* (ED I) and *emergence delirium‐nonspecific behaviors* (ED II), resulted in an excellent model fit in all indices (Table 4). The factor loadings varied between 0.95 and 0.99 for ED I and between 0.89 and 0.98 for ED II. No Haywood cases were detected. The factor correlation was 0.85 (Table 5).

**TABLE 4:** Goodness‐of‐fit indices for the ordinal confirmatory factor analyses (*n* = 347).

Model	*χ* ^2^ goodness‐of‐fit			RMSEA			CFI	TLI	SRMR
	*χ* ^2^	df	*p*	RMSEA	90% CI	*p*			
One‐factor model	93.32	5	< 0.001	0.226	0.187–0.267	< 0.001	1.00	0.99	0.06
Two‐factor model	3.87	4	0.424	0.000	0.000–0.080	0.761	1.00	1.00	0.01

Abbreviations: CFI, Comparative Fit Index (≥ 0.95); RMSEA, Root Mean Square Error of Approximation (≤ 0.06); SRMR, Standardized Root Mean Square Residual (≤ 0.08); TLI, Tucker‐Lewis Index (≥ 0.95).

**TABLE 5:** Factor loadings, residual variances, factor correlations, and reliability estimates from the ordinal confirmatory factor analyses (*n* = 347).

Items	One‐factor model	Two‐factor model	
		ED I	ED II
1. Eye contact	0.94 (0.12)	0.95 (0.10)	
2. Purposeful actions	0.99 (0.02)	0.99 (0.02)	
3. Aware of surroundings	0.96 (0.08)	0.97 (0.07)	
4. Restless	0.86 (0.26)		0.89 (0.20)
5. Inconsolable	0.92 (0.16)		0.98 (0.04)
Factor correlation		0.85	
Reliability estimates			
Ordinal alpha	0.96	0.98	0.94
Ordinal omega	1.0	0.96	0.89
Cronbach's alpha	0.93	0.96	0.87

Abbreviations: ED I, Emergence delirium‐specific behaviors; ED II, Emergence delirium‐nonspecific behaviors.

### 4.5 Internal Consistency

The PAED scale demonstrated good internal consistency reliability, with an ordinal alpha of 0.98 for ED I and 0.94 for ED II. The corresponding Cronbach's alpha values were 0.96 and 0.87. The ordinal omega also supported good internal consistency reliability in ED I (*ω* = 0.96) and in ED II (*ω* = 0.89) (Table 5).

### 4.6 Convergent Validity

The analyses supported convergent validity for the PAED scale. The Spearman correlation between the PAED scale and the FLACC scale was 0.71 (*p* < 0.001), between ED I and the FLACC scale 0.64 (*p* < 0.001), and between ED II and the FLACC scale 0.80 (*p* < 0.001).

### 4.7 Cutoff Scores

**FIGURE 1:**
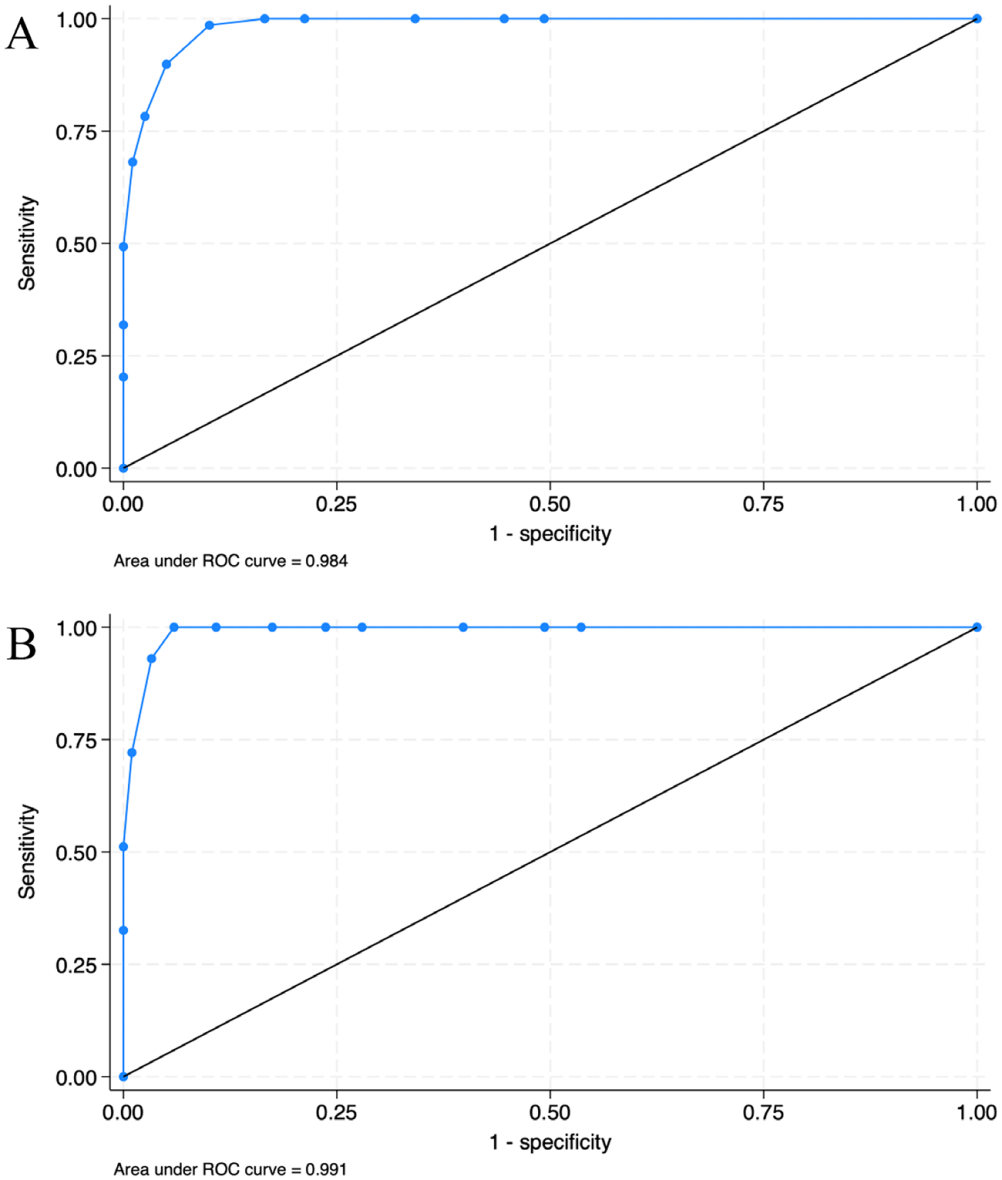
Receiver‐operating characteristic (ROC) curve analyses for ED I and Emergence Delirium (ED), (A) ED defined as PAED score ≥ 10, (B) ED defined as PAED score ≥ 13.

Using the PAED total score of ≥ 10 as the definition of ED (278 no cases vs. 69 cases), the suggested cutoff score, based on the Youden index, was ≥ 6 for ED I. Based on this cutoff score, the sensitivity and 1‐specificity were, respectively, 0.99 and 0.10, and the AUC was 0.98 (Figure 1A). When ED was defined as the PAED total score of ≥ 13 (304 no cases vs. 43 cases), the suggested cutoff score based on the Youden index was ≥ 8 for ED I. Based on this cutoff score, the sensitivity and 1‐specificity were, respectively, 1.00 and 0.06, and the AUC was 0.99 (Figure 1B).

## 5 Discussion

To the best of our knowledge, this is the first study to investigate the latent structure of the PAED scale using CFA with methods specifically designed for ordinal data. The results show that the suggested division of the PAED scale into ED I and ED II is superior to the original unidimensional model including all five items. These findings may have the potential to contribute to the accuracy of identifying children with ED among researchers and clinicians. This study also contributes by suggesting tentative cutoff scores for ED I.

In this study, the dataset exhibited high quality due to a minimal incidence of missing data. These findings indicate that the items on the PEAD scale are easy to complete, which is of importance for clinical use. Another aspect of the data quality is the score distribution. Findings from this study showed that floor effects were presented for all items and scale scores. A potential consequence of floor and ceiling effects is poor discrimination ability, i.e., reduced sensitivity and responsiveness. However, the items and scale scores are expected to exhibit floor effects as the ED behavior is only expected to occur in a minority of anesthetized children.

In the evaluation of its latent structure, the PAED scale was found to exhibit multidimensionality rather than unidimensionality. The two‐factor model showed an excellent fit in all goodness‐of‐fit indices, while the one‐factor model demonstrated poor fit in regard to the χ2 goodness‐of‐fit and RMSEA. There were also higher factor loadings in the two‐factor model than in the one‐factor model. These results are in line with previous criticism of the PAED scale concerning the delirium specificness. Locatelli et al. [16] tried to distinguish ED behaviors on the PAED scale, with consideration of the Diagnostic and Statistical Manual of Mental Disorders (DSM‐IV/5) [13] diagnostic criteria for delirium, and found the first three items (ED I) to be more delirium‐specific than the last two (ED II). Another study, which also reflected on the DSM‐IV/5 criteria, found the core behaviors for ED to be “eyes averted, stared or closed”, like Item 1 on the PAED scale, “non‐purposefulness”, like Item 2, and “nonresponsivity” [1]. One hypothesized alternative is that “nonresponsivity” could be an indirect indicator within Item 1, meaning that the absence of eye contact might contribute to communication difficulties and reduced ability to respond. Additionally, suggested behaviors for diagnosing ED were defined by Somani et al. as “no eye contact”, like Item 1 on the PAED scale, and “no awareness of surroundings”, like Item 3. Notably, “purposeful actions”, like Item 2, was excluded from the criteria, citing that only 10% of the children exhibited such behavior [24]. However, it is important to acknowledge that the dichotomous approach within that study presents a critical limitation. This limitation could lead to misinterpretation of the data, suggesting that 90% of the children exhibited unpurposeful activity, a behavior that has previously been exclusively associated with delirium. Unfortunately, this reverse concept was never considered.

In the present study, convergent validity for ED I was supported as the correlation between ED I and FLACC was weaker than that between ED II and FLACC. This indicates that ED II reflects aspects of pain rather than ED. An association between ED II and the FLACC score has been noted previously as well [6]. This explains a main part of the difficulty in distinguishing ED from pain when using the original PAED scale, which might lead to pharmacological treatment for ED when a child is suffering from pain, or vice versa. The FLACC scale is a widely used assessment tool and is more valid and reliable than the two items in ED II in identifying postoperative pain in children. Discriminant validity was not fully supported, as the factor correlation was strong (0.85). However, the factors were not completely correlated, which shows that ED I and ED II reflect two different but overlapping constructs.

In the present study, ordinal alpha and omega values above 0.95 supported internal consistency reliability of ED I. However, too‐high alpha and omega values can reflect problems with redundancy, i.e., that items capture the same thing instead of different aspects of a construct. Alpha values above 0.90 or 0.95 are commonly described as a potential problem in the literature [23]. However, if an instrument is designed to make individual decisions, like the PAED scale is, alpha values > 0.90 are commonly recommended. From this perspective, high alpha values should be required for ED I.

The suggested tentative cutoff scores for ED I were calculated to ≥ 6 or ≥ 8, depending on the cutoff value of the original five‐item PAED scale that was used as the criterium variable. In previous studies, the cutoff scores for ED I have varied between ≥ 6 and ≥ 9 [16, 17], which supports the present findings. For a clinical screening tool like ED I, high sensitivity is typically more important than high specificity. Thus, it is of utmost importance not to miss any children with ED despite the increased risk for false positive cases. From this aspect, the lower cutoff of ≥ 6 is recommended until further evidence is found.

### 5.1 Limitations

This study has some methodological limitations that need to be considered. No a priori power calculations were conducted for the study, as the evaluation of the PAED scale was only part of the larger research project. However, Brown suggests that a sample size of 150 can be appropriate for a medium‐sized CFA model using the WLSMV estimation procedure [23]. Since the PAED scale only includes five items, a sample size of 347 is probably sufficient.

The assessments with the FLACC scale were made upon awakening, but the children may still have been affected by the anesthesia and not completely awake. This may have potentially contributed to occasional inaccurate responses.

Another limitation is related to the evaluation of cutoff scores; the original five‐item PAED scale served as the criterion variable for identifying a tentative cutoff score, just as in previous studies [16, 17]. Using the same measure as a criterion variable is somewhat problematic, even if ED I only includes three of the items in the PAED. To ensure that a cutoff score is valid and reliable, it is preferable to use multiple criterion measures; otherwise, the cutoff score may not be sensitive enough to detect changes. Thus, establishing reliable cutoff scores for ED I requires further research.

## 6 Conclusions

This study contributes important evidence concerning the validity and reliability of the PAED scale. Most importantly, ED I seems to be a more valid and reliable measure than the original five‐item scale. These present findings suggest that the application of ED I may contribute to enhanced diagnostic accuracy for this condition. A three‐item version is also easier to use from a clinical perspective. The suggested tentative cutoff scores need to be further evaluated.

## Ethics Statement

The principles of the Declaration of Helsinki, as well as the national ethical guidelines for research, were followed.

## Consent

Written informed consent was obtained from the parents or guardians of the participating children.

## Conflicts of Interest

The authors declare no conflicts of interest.

## Data Availability

The data that support the findings of this study are not openly available.
